# Single-cell analysis and spatial resolution of the gut microbiome

**DOI:** 10.3389/fcimb.2023.1271092

**Published:** 2023-10-04

**Authors:** Bhoomi Madhu, Brittany M. Miller, Maayan Levy

**Affiliations:** Department of Microbiology, Perelman School of Medicine, University of Pennsylvania, Philadelphia, PA, United States

**Keywords:** microbiome, single cell, genomics, sequencing, spatial resolution

## Abstract

Over the past decade it has become clear that various aspects of host physiology, metabolism, and immunity are intimately associated with the microbiome and its interactions with the host. Specifically, the gut microbiome composition and function has been shown to play a critical role in the etiology of different intestinal and extra-intestinal diseases. While attempts to identify a common pattern of microbial dysbiosis linked with these diseases have failed, multiple studies show that bacterial communities in the gut are spatially organized and that disrupted spatial organization of the gut microbiome is often a common underlying feature of disease pathogenesis. As a result, focus over the last few years has shifted from analyzing the diversity of gut microbiome by sequencing of the entire microbial community, towards understanding the gut microbiome in spatial context. Defining the composition and spatial heterogeneity of the microbiome is critical to facilitate further understanding of the gut microbiome ecology. Development in single cell genomics approach has advanced our understanding of microbial community structure, however, limitations in approaches exist. Single cell genomics is a very powerful and rapidly growing field, primarily used to identify the genetic composition of microbes. A major challenge is to isolate single cells for genomic analyses. This review summarizes the different approaches to study microbial genomes at single-cell resolution. We will review new techniques for microbial single cell sequencing and summarize how these techniques can be applied broadly to answer many questions related to the microbiome composition and spatial heterogeneity. These methods can be used to fill the gaps in our understanding of microbial communities.

## Introduction

The human organism is colonized by a vast community of microorganisms, which support and maintain many aspects of our health. The intestinal microbiota contributes to multiple physiological functions of the host, including metabolic homeostasis, immunity, and neuronal activity. In turn, the host provides a stable colonization niche for commensal microorganisms and ensures continuous influx of dietary nutrients. Despite advances in sequencing and culturing techniques, a large amount of undiscovered and otherwise undescribed microbial taxa remains unknown, including in the human gut (Almeida et al., 2019). The intestinal microbiota has been implicated in the etiology of a variety of human diseases, including those localized to the gastrointestinal tract such as inflammatory bowel disease (IBD) (Glassner et al., 2020), Crohn’s Disease (Pascal et al., 2017), susceptibility to pathogenic bacterial infection (Ivanov et al., 2009; Theriot et al., 2014; Velazquez et al., 2019), as well as a number of other extraintestinal diseases like cardiovascular diseases (Jie et al., 2017; Yoo et al., 2021), depression (Limbana et al., 2020) and obesity (Liu et al., 2021). Research efforts are focusing on exploring causality between changes in the gut microbiota and disease, with the aim to improve understanding to lead to therapeutics as well as more robust prevention strategies. Sequencing techniques are being continually refined, with a current goal being the focus on sequencing at the single cell level, rather than bulk sequencing, in order to gain finer resolution and understanding of microbial communities.

Advancements in technologies and techniques in the field of DNA sequencing, particularly with regards to the gut microbiota, have added to our understanding of the role of the microbiota in human health and disease. However, until recently, the study of the gut microbiota was limited to the study of those microbes which could be isolated and cultured. The usage of ribosomal RNA genes as a classification system for microbes, along with Sanger sequencing which allowed for the automated sequencing of DNA in the late 1970s, set the stage for the study and classification of a number of microbes, culturable or not (Escobar-Zepeda et al., 2015). Improvements made to Sanger sequencing method, such as the replacement of radio isotopes with the use of fluorometric based detection methods, as well as detection via capillary-based electrophoresis lead to the development of the first DNA sequencing machines in the late 1980s and early 1990s, allowing for the sequencing of bacterial and other more complex genomes (Heather & Chain, 2016). Sequencing based on the Sanger method, or dideoxy method, was prevalent for a number of years, until second-generation DNA sequencing technology was developed. Pyrosequencing, unlike Sanger sequencing, does not require the use of labeled dideoxy nucleotides and subsequent visualization, rather, it takes advantage of an enzymatic reaction by which light is produced proportional to the amount of nucleotide binding (Heather & Chain, 2016). The advent of pyrosequencing, like Sanger sequencing, revolutionized the field, as now sequencing reactions could be run in parallel with near instant results. Later, Ion Torrent sequencing was developed, which does not require the use of fluorescence or luminescence, but rather measures nucleotide incorporation by the change in pH cause by the proton release during polymerization, allowing for very rapid sequencing (Heather & Chain, 2016). However, one of the most commonly used second-generation DNA sequencing technologies is the Illumina next generation sequencing system. The Illumina sequencing platform uses sequencing by synthesis methodology where sequencing takes place in multiple cycles that capture the fluorescence signal emitted when a correct base is added to the growing DNA strand (Gloor et al., 2010). Presently, third-generation technologies are being developed with the aim of having longer read length capabilities with lower cost, however, for many researchers, second-generation sequencing techniques are most often used (Escobar-Zepeda et al., 2015). One such third-generation sequencing technology is Pacific Biosciences’ single molecule, real-time sequencing technology (SMRT) that generates raw reads longer than 10kb in length and is popular for sequencing complex microbial communities (Tsai et al., 2016; Sadowsky et al., 2017; Li et al., 2021).

Investigation of the microbiome primarily relies on meta-omics, or the analyses of microbial DNA, RNA, or metabolites recovered from samples. The most common method used by researchers today is the 16S rRNA gene-amplicon sequencing (Tolonen and Xavier, 2017). However, this method and other commonly used sequencing techniques have drawbacks, including taxonomic blind spots, as well as the loss of information of low abundance members of the microbiota (Hatzenpichler et al., 2020; Bowers et al., 2022). The advent of single cell isolation and sequencing have been crucial to addressing this issue, however, there still remain further hurdles to the application of single cell technology for the analysis of gut microbiota. Many technical issues are being addressed by advances in technologies which will be described here. Here, we will discuss new technologies and techniques for the sequencing of the human microbiome, including the spatial aspect of the microbial community.

## Single cell isolation methodologies

**Figure 1:**
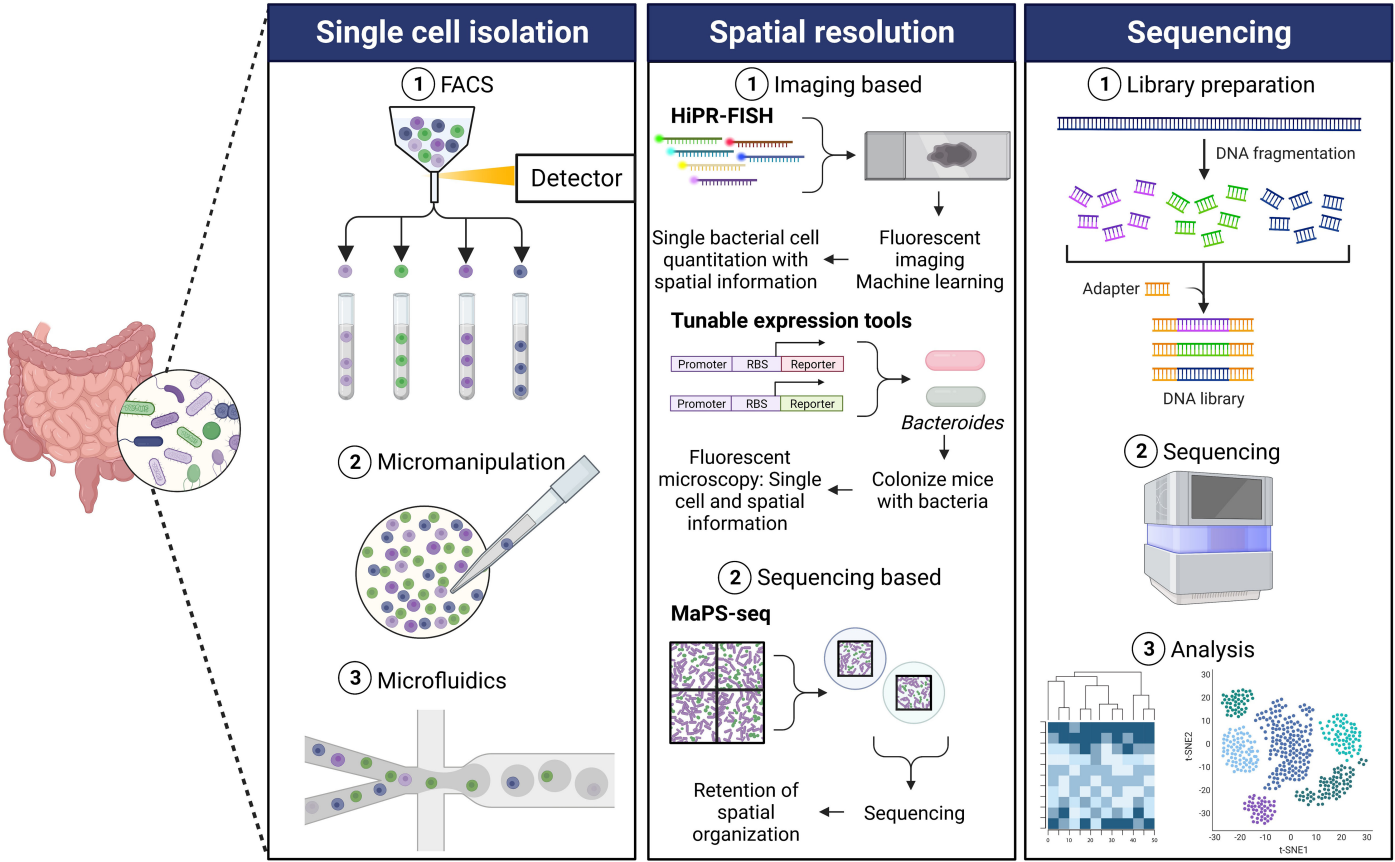
Single cell sequencing workflow. Single cell isolation: The first step for performing single cell microbial genomics is isolating single cells. Some commonly used single cell isolation techniques include fluorescence-activated cell sorting (FACS), micromanipulation, and microfluidics. (1) FACS involves size- and fluorescence-based separation that separates single cells from a complex microbial community (Rinke et al., 2014). (2) The traditional micromanipulation method includes micro-pipetting in combination with an inverted microscope for the isolation of single cells from a mix of microbial cells (Ishøy et al., 2006). (3) The microfluidics approach combined with droplet encapsulation involves encapsulating individual cells in hydrogel microspheres resulting in isolated single cells in each droplet (Marcy et al., 2007). There have been different modifications of the microfluidics method, based on the core concept of encapsulating single cells in droplets, to yield single cells. Spatial resolution: Various new techniques have been developed in order to obtain spatial genomics information of the gut microbiota, generally either imaging based or sequencing based. (1) High phylogenetic resolution fluorescence *in-situ* hybridization (HiPR-FISH) employs a binary barcode system based on hybridization of distinct fluorophores (Shi et al., 2020). Spectra are measured using fluorescence microscopy, and spectral barcodes are decoded using machine learning. Identification and spatial visualization of taxa are possible. Tunable expression tools (Whitaker et al., 2017) is a platform for engineering Bacteroides using a novel phage promoter and translation tuning strategy to enable imaging of fluorescent bacteria. Unique fluorescent signals can be used to allow differentiation of species within the gut. (2) Metagenomic plot sampling by sequencing (MaPS-seq) combines genomic and spatial resolution (Sheth et al., 2019). Intact microbiota samples are fractured into particles and are encapsulated in droplets before deep sequencing. This results in the retention of spatial information and can identify species that tend to co-localize in complex samples such as the gut microbiota. Sequencing: (1) The sequencing step of the single cell genomics workflow involves using DNA from the isolated cells to prepare a library. (2) This is followed by high-throughput sequencing which yields (3) critical information identifying the genetic composition microbes and associated gene expression changes in a complex microbial community.

Sequencing the microbiome at a resolution of individual microbes has been recently gaining popularity. There has been substantial development for single cell genomics approaches for prokaryotes and eukaryotes, enhancing the feasibility of such experiments for researchers. However, single cell sequencing from microbial communities poses challenges unique to microbes that are not considered for mammalian single cell sequencing. A few limitations with isolating single microbial cells include aggregation of bacteria which makes it difficult to isolate single cells efficiently (Trunk et al., 2018); bacterial cell walls pose a challenge for many single cell sequencing approaches and therefore require to be permeabilized (Blattman et al., 2020; KuChina et al., 2021); low biomass and low abundance of mRNA (Blattman et al., 2020; KuChina et al., 2021). For these techniques, the first step involves isolation of single cells (**Figure 1**). Despite the progress in the field of genomics, limitations exist for the first step of isolating single cells, an important part of the workflow to perform high-throughput single cell genomics. Several widely used methods of isolating single cells include fluorescence-activated cell sorting (FACS), micromanipulation, and microfluidics (Blainey, 2013; Tolonen and Xavier, 2017).

FACS is one of the most commonly used high-throughput methods for isolation of individual cells (Stepanauskas and Sieracki, 2007). Microbial cells can be individually sorted on the basis of their size and fluorescence by FACS (**Figure 1**). Rinke and colleagues described a protocol to isolate single cells from environmental microorganisms using FACS followed by extraction and amplification of their genomes (Rinke et al., 2014). A key advantage of using FACS to isolate single cells is minimized risk of contamination by extracellular DNA because of the low volume requirement. Other advantages of using FACS as the method to separate individual cells for genomic sequencing are that it has high throughput, can be automated, and is compatible with downstream applications. On the other hand, some limitations of using FACS for single cell genomics are the inability to reduce reaction volumes to nanogram range, increased caution necessary to avoid external contamination during open-plate workflows, and lack of ability to inspect cells visually (Stepanauskas, 2012; Rinke et al., 2014; Hu et al., 2016; Woyke et al., 2017).

In addition to the use of FACS, traditional methods of micromanipulation that include using micro pipetting combined with an inverted microscope as a visual aid have been used to isolate single bacterial cells (**Figure 1**) (Stepanauskas, 2012; Hu et al., 2016; Woyke et al., 2017). Using this approach, individual cells are selected and physically delivered to be processed for downstream applications. Micromanipulation has been used to capture single microbial cells from different bacterial habitats including a low pH and high temperature hot spring to study the microbial ecology in their natural environments (Ishøy et al., 2006). Hohnadel and colleagues developed an improved micromanipulation method to isolate and detect single microbial cells in food samples (Hohnadel et al., 2018). Automated versions of this method to select single cells using capillary micropipettes and associated robotics have been developed and used for bacterial single cell gene expression analysis (Anis et al., 2008; Gao et al., 2011). Major drawbacks of this approach are that it is extremely time-consuming, labor-intensive, and low-throughput (Blainey, 2013; Chen et al., 2017). Another limitation associated with this method is the risk of contamination from the laboratory environment, equipment, and RNA contamination (Blainey, 2013; Hodne and Weltzien, 2015; Chen et al., 2017).

Another widely growing technique for isolating single cells for the downstream application of genomics is the microfluidics method (**Figure 1**). Microfluidics was one of the first methods used for cell isolation for microbial single cell studies (Marcy et al., 2007; Leung et al., 2012). Key advantages of microfluidics include the ability to visualize target cells and reduce reaction volumes. The microfluidics approach provides the benefits of high-throughput isolation and barcoding individual genomes (Leung et al., 2012; Chen et al., 2017; Woyke et al., 2017). Newer techniques involving a combination of microfluidics and single cell encapsulation in droplets are rapidly growing. The droplet microfluidics method involves encapsulating single cells in hydrogel microspheres or generating water-in-oil droplets. This is followed demulsifying the droplets followed by sequencing (Tauzin et al., 2020; Pryszlak et al., 2022) or by fragmenting and barcoding individual genomes from the cells in each droplet, enabling pooled sequencing of many tagged genomes simultaneously (Zengler et al., 2002; Hosokawa et al., 2017; Woyke et al., 2017). Lan and colleagues successfully used gel microdroplets combined with microfluidics to perform single cell genomics of a synthetic community of Gram-negative and Gram-positive bacteria (Lan et al., 2017). Lim and colleagues developed PCR-Activated Cell Sorting (PACS) that utilizes the microfluidic droplet method to encapsulate individual bacteria in picoliter volume droplets which are then subjected to TaqMan PCR to identify bacteria of interest (Lim et al., 2015). These can then be used for downstream applications including genome sequencing. This method offers the advantage of performing single cell genomics in complex ecosystems.

## Bacterial single cell whole genome sequencing

Bacterial single cell whole genome sequencing field is advancing rapidly. There are many technologies that have contributed to the efficient characterization of microbes at a single cell resolution. Chijiiwa et al. reported identification of gut bacteria that responded to dietary fiber by using a novel single cell genome sequencing method (Chijiiwa et al., 2020). This method includes single cell isolation of gut microbes by capturing them in agarose gel beads by a microfluidic droplet generator. This was followed by amplification of single cell amplified DNA, captured into the gel beads, as a single cell amplified genome (SAG) library. The SAG-gel platform allows for the specific sequencing of researcher-selected samples out of the large numbers of SAGs and is also cost-efficient. Lan et al., described a high-throughput single-cell genomic sequencing (SiC-seq). SiC-seq utilizes droplet microfluidics to capture individual microbial cells in microgels. This is followed by cell lysis, DNA fragmentation and barcoding, pooling tagged DNA fragments, and sequencing. The workflow of SiC-seq was validated using an artificial microbial community comprising of yeast, Gram-negative bacteria, and Gram-positive bacteria (Lan et al., 2017). A newer high-throughput single cell sequencing method called Microbe-seq was developed by Zheng and colleagues. The Microbe-seq methodology yielded a large number of individual microbial genomes-without culturing-from longitudinally collected human gut microbial samples. This technique involves encapsulating individual microbes in droplets using a microfluidics platform followed by performing whole-genome amplification and barcoding DNA within the droplets, generating multiple SAGs per sample. The tagged DNA is then pooled and sequenced. The SAGs obtained from the Microbe-seq were then co-assembled to yield strain-level resolution of the gut microbiota samples (Zheng et al., 2022). However, techniques such as SiC-seq and Microbe-seq offer low recovery of genomes. Hosokawa and colleagues used the SAG-gel technology to recover high-quality, near complete bacterial genomes from propidium monoazide-treated human gut microbiome samples (Hosokawa et al., 2022). Arikawa et al., integrated single cell genomics and metagenomics to create a single-cell metagenomics workflow to improve the recovery of strain-resolved genomes from human microbiota samples which yielded high-quality recovery of nearly complete microbial genomes (Arikawa et al., 2021). Jin and colleagues developed a high-throughput method called Barcoding Bacteria for Identification and Quantification (BarBIQ) that provides abundance and identification at an individual microbe resolution. BarBIQ involves encapsulation of barcoded-single cells in droplets and uses 16s rRNA sequences to classify and quantify individual microbes into cell-based operational taxonomy units. This study validated the workflow of BarBIQ by comparing the effect of vitamin A deficiency on proximal and distal cecal microbiota abundance and composition in mice (Jin et al., 2022). One limitation of SAGs is that they have incomplete sequences because of the introduction of biased sequencing during amplification cycles. To address this, Kogawa and colleagues developed a single-cell amplified genome long-read assembly workflow that enables construction of complete SAGs using long reads (Kogawa et al., 2023).

Another technique for single cell genomic sequencing used by investigators is the single droplet multiple displacement alignment (sd-MDA) method. This technique also involves the encapsulation of single cells in droplets followed by whole genome alignment (WGA). Single cells are passed through a droplet generator. Hosokawa and colleagues successfully used this method for single cell genome sequencing involving both bacterial cells and human cancer cells. Some advantages of this method include increased efficiency of sample preparation and reduced cost and labor investment (Hosokawa et al., 2017). Despite being indispensable for whole-genome sequencing, there are some limitations of this method associated with MDA. The limitations include low coverage due to genome coverage bias and potential DNA contamination (Džunková et al., 2014; Hosokawa et al., 2017). Advances in the isolation of single cells have made downstream single cell sequencing possible.

## Bacterial single cell transcriptomics

Single cell sequencing has tremendously helped in studying phenotypic heterogeneity of microbes. It has also enabled exploring rare organisms using high throughput single cell analysis (KuChina et al., 2021). A number of new technologies have emerged which have refined single-cell transcriptional analysis of microbes, especially in mixed and complex communities like the human gut microbiome.

One of the recently developed single cell transcriptomics that can be applied to microbes is split-pool ligation-based transcriptome sequencing (SPLiT-Seq). SPLiT-seq overcomes the need for microfluidics as it utilizes a combinatorial barcoding approach. In this method, cells are distributed in individual wells and barcoded primers are used to synthesize cDNA through intracellular reverse transcription. This step is followed by multiple rounds of pooling and splitting accompanied by barcoding in every round. Finally, the reads are combined by referring to the barcode combination to assemble the transcriptome. An advantage of this technique is that it circumvents the need of isolating single cells, and it enables scalable multiplexing (Rosenberg et al., 2018). Kuchina and colleagues developed a modified version of SPLiT-seq tailored to identify microbial subpopulations and associated gene expression changes, called microSPLiT (microbial split-pool ligation-based transcriptomics). Transcriptional responses to heat shock exposure to *Escherichia coli* and *Bacillus subtilis* were reliably detected using microSPLiT. Furthermore, this technique also captured signature transcriptional changes through the growth cycle of *B. subtilis*. Transcriptional changes in stress responses, regulation of carbon utilization, developmental decisions, and metal uptake were revealed through microSPLiT, indicating its capabilities of identifying heterogeneity in cellular and regulatory pathways. Together, these analyses demonstrate the potential of microSPLiT for detecting differential gene expression associated with heterogeneous cell populations in varied environments (KuChina et al., 2021).

Another recently developed high throughput prokaryotic single cell RNA-seq (scRNA-seq) technique is prokaryotic expression profiling by tagging RNA *in situ* and sequencing (PETRI-Seq) (Blattman et al., 2020). PETRI-seq uses an *in situ* combinatorial indexing approach to barcode bacterial transcripts. The methodology of PETRI-seq involves fixation and permeabilization of cells, split-pool barcoding, and library preparation for sequencing. Blattman and colleagues developed PETRI-seq and successfully used it for high-throughput sequencing *E. coli* and *Staphylococcus aureus* with high single cell purity. The authors demonstrated that PETRI-seq enabled successful distinction between *E. coli* populations in different growth phases. This was achieved by using complementary approaches of comparing operon expression patterns and Gene Ontology terms associated with exponential and stationary phases. PETRI-seq, owing to its high throughput capacity, detected a rare subpopulation of *S. aureus* undergoing prophage induction by applying principal component analysis to 6,663 single cell transcriptomes of *S. aureus* (Blattman et al., 2020).

A FACS-based bacterial scRNA-seq workflow was developed by Imdahl et al. which utilizes poly(A)-independent multiple annealing and dC-tailing-based quantitative scRNA-seq (MATQ-seq) protocol. The bacteria are sorted into single cells using FACS and are then enzymatically lysed. The MATQ-seq protocol is used to obtain cDNA from individual bacterial cells followed by tagmentation and library preparation to generate libraries for sequencing. The study used this scRNA-seq workflow to characterize global transcriptomes of *Salmonella* populations under different growth conditions resolved at a single cellular level (Imdahl et al., 2020). The same group recently developed an improved version of this workflow that resulted in improved gene detection limit and coverage at a single bacterial cell resolution. This version of scRNA-seq involves the use of a more efficient reverse transcriptase and has a Cas9-based rRNA depletion step integrated in the workflow (Homberger et al., 2023).

Another FACS-based scRNA-seq was developed by Nishimura and colleagues which incorporates RamDA-seq in its workflow. The methodology involves FACS-based isolation of single bacterial cells followed by cell lysis. The workflow includes library construction using the RamDA-seq technique which is a full-length RNA sequencing method. Before sequencing, the amplified cDNA libraries are then depleted of rRNA using Cas9. The study used this hybrid scRNA-seq approach to reveal heterogeneity in different growth stages of live *E. coli* and in heat-shocked *E. coli* populations in about a quarter of *E. coli* genes at a single cell resolution (Nishimura et al., 2023).

An additional high-throughput scRNA-seq technique that has been successfully used for bacterial populations is the BacDrop that is droplet-based. The workflow of BacDrop involves fixation and permeabilization of cells followed by rRNA and gDNA depletion. The next step is generation of barcoded cDNA by reverse transcription which is then followed by capturing single cells in droplets and droplet barcoding. The last step is library preparation for sequencing. The group used BacDrop to characterize previously unknown heterogeneity based on mobile genetic elements in *Klebsiella pneumoniae* at a single cell resolution. However, the working efficiency of BacDrop remains to be validated on more complex microbial communities involving unknown genomes (Ma et al., 2023).

Together, these methodologies of single cell isolation and single cell sequencing offer the flexibility to choose the best suited approach of isolating and sequencing single cell genomes of bacteria, depending on the need of experiments.

## Gut biogeography through the lens of community analysis

Single cell sequencing at the base level does not maintain information about where in space the cells were located in the original sample, but newer methods have been applied to tackle this problem.

The spatial localization of pathogens within the host has been understood as a key factor to their pathogenesis, however, less is understood about the localization of commensals, and how the change in localization affects health and disease (Lopez et al., 2016; Spiga and Winter, 2019; Rogers et al., 2021). Localization of gut microbiota members along the gastrointestinal tract differs in both cross-sectional (from epithelium and mucus-associated to the lumen) and longitudinal (from stomach to distal colon) heterogeneity, the latter of which does not require novel technologies to study, as researchers can sample from different locations longitudinally along the GI tract (Donaldson et al., 2016; Tropini et al., 2017). However, identification of localization of gut microbiota members in the cross-sectional aspect is more difficult to address. There are many factors known to influence not only the gut microbiota, but also the spatial distribution of the members of the microbiota, such as oxygen and ROS species (Albenberg et al., 2014; Lopez et al., 2016; Miller et al., 2020), the physical barrier mucus (Van der Sluis et al., 2006; Johansson and Hansson, 2016), pH (O’May et al., 2005; Ilhan et al., 2017), various immune effectors produced by the host, such as antimicrobial peptides (Bevins and Salzman, 2011), and availability of nutrients (Koropatkin et al., 2012). Additional habitat filters varying both longitudinally and cross-sectionally include electron acceptor availability (Miller et al., 2021; Liou et al., 2022).

Research on changes in the microbiota has often been focused on large changes in community composition associated with disease, especially with the growth of certain pathogenic species. It is now becoming better appreciated that changes in the spatial distribution of species in the gut is also associated with disease and infection, as well as several chronic conditions, such as IBD (Swidsinski et al., 2005), and colorectal cancer (Dejea et al., 2014; Saffarian et al., 2019), and various other perturbations such as starvation, antibiotics, and surgery (Zaborin et al., 2020). It is therefore necessary for analysis of gut microbial communities to move beyond defining the composition of the microbiota to a spatial understanding, to better probe questions about microbiota function and interaction between other species and the host. Answering questions about how certain members of the microbiota are interacting with both the host as well as other microbiota members will shed light on a variety of human diseases, as well as help define homeostasis and dysbiosis of the gut, and moving towards causation of the later (Tiffany and Bäumler, 2019).

High-throughput -omics techniques have revolutionized the field of gut microbiota research. Much work has relied on the use of fecal samples, whose use is pervasive due to the ease of sample collection, however, fecal samples usage faces drawbacks such as decay of microbes, and the loss of information about spatial differences in community members, both along the GI tract and in the cross-sectional plane. Many genomic techniques rely on a homogenized sample, which therefore results in the loss of important spatial information in the larger picture of the host-microbiota environment. Laser Capture Microdissection (LCM) has provided a method of high-resolution site-specific sampling of microbial communities (Espina et al., 2006). Using LCM, researchers can select a sample region with use of a microscope, capturing the specific area of interest, and the sample can be used downstream for metagenomic or other analyses. Several studies have delved into newer technologies providing single-cell spatial genomic information. Generally, these technologies can be classified as image-based or sequencing-based.

## Spatial resolution of DNA sequencing

The use of *in situ* hybridization (ISH), an image-based approach, has been available to researchers for a number of years (Rudkin and Stollar, 1977). ISH depends on labeled probes hybridizing specific DNA or RNA targets, often employing use of a fluorophore, as in fluorescent *in situ* hybridization (FISH), which allows for the location of the target of interest under the microscope. A limitation of FISH in microbiota samples is the limited number of fluorophores available for visualization and discrimination of different targets. Advances in the field of RNA-FISH have alleviated issues with multiplexity and other problems with FISH, such as the analysis of targets with low copy numbers and could be applied to DNA targets in the microbiota in the future (Eng et al., 2019).

A recently developed method known as High Phylogenetic Resolution FISH (HiPR-FISH) combines FISH, a visual assay targeting ribosomal RNA for visualization and identification, with a binary barcoding scheme and machine learning of fluorophore combinations (**Figure 1**) (Shi et al., 2020). This method provides a microbial mapping technology that can identify various taxa with aid of a microscope. Using ten fluorophores, probes with the same encoding sequence, but different readout sequences, can bind in equal amounts to ribosomal RNA (rRNA) molecules within the same cell. As bacterial cells contain hundreds of 16S rRNA copies, each species can be targeted by encoding probes targeting the same sequence, but flanked by different readout sequences, allowing for the assignment of a unique combination of fluorophores. The fluorescence emission spectra are measured, and a spectral barcode is computed. The spectra recorded on each pixel is averaged, and a machine learning classifier decodes the cell barcodes. The authors were able to achieve single-cell quantitation and found previously undescribed genera in the human oral plaque microbiome (Shi et al., 2020). The authors additionally applied this novel method to study the effect of antibiotics on the spatial organization of the gut microbiome, targeting up to 47 genera using HiPR-FISH, and revealed spatial association disruption between several genera. The authors demonstrate single-cell mapping of complex communities, which allows for bacterial spatial organization questions to be addressed.

Another image-based method described recently by Whitaker et al. (2017) uses a phage promoter system to introduce genomically integratable vectors into *Bacteroides* in a high-throughput fashion, using an adaptation on the Golden Gate cloning method (**Figure 1**). This method allows for the *in vivo* imaging of fluorescent *Bacteroides*, as the fluorescent proteins were expressed at higher levels than achievable previously using other promoters, such as the 16s rRNA promoter. Importantly, the authors show this does not come at a fitness cost for the bacteria *in vivo*. Their technique allowed for the differentiation of each species at a single-cell level, using unique combinations of GFP and mCherry expression. The authors use this technique to visualize *Bacteroides* in colonic crypts of the mouse gut and show that colonizing a mouse with one isogenic strain of *B. thetaiotaomicron* provides colonization resistance against a sequentially gavaged isogenic strain. This study provides a great example of how new techniques can be used to provide strain-level resolution of spatial heterogeneity. While not a sequencing technique, this method can give insight into where a particular gut microbiota member is localized, and for the use of resolution of localization of several strains of the same microbe, as shown by the authors.

A recently described sequencing-based spatial technology, termed Metagenomic Plot Sampling by Sequencing (MaPS-seq), is an unbiased technique that avoids the use of a microscope that does not involve single cell analysis, and instead analyzes the sequences of microbial cells in their native geographical context (**Figure 1**) (Sheth et al., 2019). An input sample is first fixed and embedded into a polymer matrix. The matrix is then fractured into clusters, the cells are lysed, and clusters of desirable size are selected. This process ensures that the clusters contain the genomic DNA immobilized in the original spatial location, preserving this information. The clusters are then encapsulated with barcoded beads, containing the 16s rRNA amplification primers. The primers are cleaved from the beads, genomic DNA is released, and PCR amplification of the 16s V4 region is performed. Droplets are separated, and the library is deep sequenced. The authors applied MaPS-seq to analyze spatial metagenomics of the mouse colon microbiome and found that certain community members aggregate or clump together across the distal colon. They then use MaPS-seq to investigate the spatial organization along the GI tract and found that certain taxa tended to be found close to each other, or co-associate, such as *Lactobacillaceae* and *Lachnospiraceae*. Further investigation into the benefit of co-associations of bacteria that tend to aggregate together spatially in the gut will be illuminating to the field and could help uncover factors necessary for the culture of certain currently unculturable microbes. MaPS-seq also has the potential to profile interactions between bacteria and eukaryotes, such as fungi or epithelial cells, by modifying the capture primers, as noted by the authors.

While further new technique development is necessary, many cutting-edge techniques for capturing spatial information have been developed recently, as described here. In addition to the techniques described that have been applied to the gut microbiota, several spatial transcriptomics tools have been described in the study of mammalian tissues, especially with regards to the brain. Multiplexed mRNA-FISH has been applied to spatial mapping of mRNA molecules, with the washing and adding fluorophores, to image the same section multiple times for different targets, achieving high multiplexity (Goh et al., 2020). Slide capture technology, a technique where mRNA molecules are hybridized to DNA anchors on a slide, followed by reverse transcription and sequencing, has been applied to the mouse brain to reveal responses to traumatic brain injury (Rodriques et al., 2019), and in the study of breast cancer tissues (Vickovic et al., 2019). However, as this method relies on the polyadenylation of mRNA for capture, it cannot be used for the spatial mapping of microbes, as microbes generally do not have polyadenylated mRNAs. Another method described recently that proves more promising for the gut microbiota field, DNA Microscopy, is a microscope-free method by which transcripts are tagged with randomized nucleotides, followed by amplification tags and concatenation of the copies (Weinstein et al., 2019). A computer algorithm then decodes proximities based on these concatenated sequences and infers an image of the original transcripts at cell-level resolution. DNA Microscopy could theoretically be applied to mapping the spatial relationship of microbes in the gut microbiota but has yet to be applied in practice.

## Discussion

High-resolution single cell analysis for microbes allow capturing heterogeneity in bacterial populations identifying rare subpopulations of bacteria, which might be missed in traditional bulk sequencing (KuChina et al., 2021). Various methodologies have been developed to isolate single eukaryotic cells which have been optimized for bacterial cells. This allows performing single cell sequencing and is of great value for in-depth characterization of the microbiome as well as host-microbiome interactions (Woyke et al., 2017). Powerful high throughput sequencing of single microbial cells allows researchers to address questions that will provide insights into their genomes and spatial function of gut microbiome in the etiology of gut and other related diseases (Tolonen and Xavier, 2017; Sharma and Thaiss, 2020). Sequencing gut bacteria at the level of single cells better captures the heterogeneity compared to traditional bulk sequencing and enables the study of microbes that make up a small fraction of a population. This could aid with the tracking emergence of drug resistance in bacteria (Tolonen and Xavier, 2017).

Resolving gut microbiome at the single cell level opens the possibility of addressing new scientific questions. A cross sectional study showed that immunocompromised patients, particularly children with sickle cell disease (SCD) receiving penicillin prophylaxis in the cohort, showed differences in alpha diversity and bacterial abundance of the gut microbiota compared to patients not receiving penicillin (Mohandas et al., 2020). Single cell resolution of gut microbiota will provide a closer look at their functional implications in SCD and other immunodeficiency diseases.

Gut microbiota composition and function are heavily influenced by environmental factors, diet, and age. Studies report patterns of gut microbes that not only influence aging but can also predict age-associated decline (Bosco and Noti, 2021). It will be of interest to identify the effect of diet and age on gut microbiome in immunocompromised patients. The technological advancements in resolving gut microbiota at the level of single cells will allow dissecting functional relationships between gut microbiome and immunocompromised host and how it is influenced by intrinsic and extrinsic factors such as age and diet.

In addition to being impacted by host and environmental factors, the gut microbes also influence each other. These inter-microbial interactions can be effectively captured by the genomics tools optimized for microbial communities. This will facilitate studying how the microbes impact each other and shape the gut microbiome (Bäumler and Sperandio, 2016; Coyte and Rakoff-Nahoum, 2019).

## Author contributions

ML: Supervision, Writing – review & editing. BM: Writing – original draft, Writing – review & editing. BMM: Writing – original draft, Writing – review & editing.
